# Identification of a Common Conformational Epitope on the Glycoprotein E2 of Classical Swine Fever Virus and Border Disease Virus

**DOI:** 10.3390/v13081655

**Published:** 2021-08-20

**Authors:** Yu-Liang Huang, Denise Meyer, Alexander Postel, Kuo-Jung Tsai, Hsin-Meng Liu, Chia-Huei Yang, Yu-Chun Huang, Nicholas Berkley, Ming-Chung Deng, Fun-In Wang, Paul Becher, Helen Crooke, Chia-Yi Chang

**Affiliations:** 1 OIE Reference Laboratory for Classical Swine Fever, Animal Health Research Institute, Council of Agriculture, Executive Yuan, 376 Chung-Cheng Road, Tansui, New Taipei City 25158, Taiwan; ylhuang@mail.nvri.gov.tw (Y.-L.H.); krtsai@mail.nvri.gov.tw (K.-J.T.); hmliu@mail.nvri.gov.tw (H.-M.L.); chyang@mail.nvri.gov.tw (C.-H.Y.); ychuang1@mail.nvri.gov.tw (Y.-C.H.); mcdeng@mail.nvri.gov.tw (M.-C.D.); 2 EU and OIE Reference Laboratory for Classical Swine Fever, Institute of Virology, University of Veterinary Medicine Hannover, 30559 Hannover, Germany; denise.meyer@tiho-hannover.de (D.M.); alexander.postel@tiho-hannover.de (A.P.); paul.becher@tiho-hannover.de (P.B.); 3 OIE Reference Laboratory for Classical Swine Fever, Animal and Plant Health Agency, New Haw KT15 3NB, Surrey, UK; Nick.Berkley@apha.gov.uk; 4 School of Veterinary Medicine, National Taiwan University, No. 1, Section 4, Roosevelt Road, Taipei 10617, Taiwan; fiwangvm@ntu.edu.tw

**Keywords:** classical swine fever virus, border disease virus, glycoprotein E2, epitope mapping, cross reactivity, conformational epitope, attachment, entry

## Abstract

Classical swine fever virus (CSFV) shares high structural and antigenic homology with bovine viral diarrhea virus (BVDV) and border disease virus (BDV). Because all three viruses can infect swine and elicit cross-reactive antibodies, it is necessary to differentiate among them with regard to serological diagnosis of classical swine fever. To understand the mechanism of cross-reactivity, it is important to define common or specific epitopes of these viruses. For this purpose, epitope mapping of six monoclonal antibodies (mAbs) was performed using recombinant expressed antigenic domains of CSFV and BDV E2 proteins. One CSFV-specific conformational epitope and one CSFV and BDV common epitope within domain B/C of E2 were identified. Site-directed mutagenesis confirmed that residues G725 and V738/I738 of the CSFV-specific epitope and P709/L709 and E713 of the second epitope are important for mAbs binding. Infection of CSFV in porcine cells was significantly reduced after pre-incubation of the cells with the domain B/C of E2 or after pre-incubation of CSFV with the mAbs detecting domain B/C. 3D structural modeling suggested that both epitopes are exposed on the surface of E2. Based on this, the identified epitopes represent a potential target for virus neutralization and might be involved in the early steps of CSFV infection.

## 1. Introduction

Classical swine fever virus (CSFV; *Pestivirus C*) is the etiological agent of classical swine fever (CSF), which is a highly contagious disease of swine. CSFV is an enveloped positive-stranded RNA virus, which together with bovine viral diarrhea virus (BVDV) type-1 (*Pestivirus A*) and type-2 (*Pestivirus B*), border disease virus (BDV; *Pestivirus D*), and a growing number of new species belongs to the genus *Pestivirus* of the family *Flaviviridae* [1,2]. The genome of CSFV is approximately 12.3 kb in length, is flanked by 5′ and 3′ non-translated regions, and contains one large open reading frame (ORF). The single ORF encodes for a polyprotein, which is co- and post-translationally processed by viral and cellular proteases into twelve viral proteins, including four structural proteins, namely, the capsid protein (C) and the three envelope (E) glycoproteins E^rns^, E1, and E2 [3]. CSFV infection is limited to swine, while BVDV and BDV infect both ruminants and swine. CSFV shares high structural and antigenic homology with BVDV and BDV [4]. Infections of swine with ruminant pestiviruses can result in the production of cross-reacting antibodies, which can cause problems within the serological diagnosis of CSF [5]. In the context of former CSF sero-surveillance studies conducted in the Netherlands and in Japan, the BDV strains Frijters and FNK2012-1 were detected in pigs due to cross reactivity in CSFV-specific assays, respectively [6,7]. Moreover, it has been described that the Aydin pesitivirus and the novel ovine pestivirus from Italy are more closely related to CSFV than to BDV, resulting in significant interference with the serological diagnosis of CSF [8,9,10,11].

The envelope glycoprotein E2 is the immunodominant protein of pestiviruses. E2 is the major protein exposed on the outer surface of the virions and induces neutralizing antibody responses during virus infection [12]. The E2 of pestiviruses plays several important roles during the viral life cycle [13], such as interacting with cellular receptors to facilitate virus attachment and mediating membrane fusion to allow entry into host cells [14,15], and thereby determines the cell tropism and host specificity of pestiviruses [16,17]. The sequences and structures of E2 proteins are presumed to be responsible for the host specificity with regard to cell entry [18,19] through receptor-mediated endocytosis [20,21,22]. Bovine CD46 is the cell surface receptor for BVDV [23], and inhibition of BVDV infection by CSFV E2 suggests that CSFV E2 and BVDV E2 share an identical receptor [14]. Furthermore, it has been reported that both porcine CD46 and heparan sulfates are the major factors for CSFV attachment and entry [24]. However, recent experiments with porcine CD46 knockout cells clearly showed that porcine CD46 is not the major receptor for CSFV, but for atypical porcine pestivirus (APPV) [25].

For CSFV, the antigenic structure of E2 and its epitopes have been extensively studied. The four antigenic domains defined for the E2 protein are located in the N-terminal half and are arranged in the order of B/C/D/A [26,27]. The domains B/C and D/A represent two independent structural units [28]. The conformational epitopes of E2 depend on the pairing of six cysteine residues to achieve the correct folding of these four domains [28,29,30]. Epitopes of domain B/C are mainly not conserved among the different CSFV genotypes and thus are responsible for antigen specificity, whereas the protein sequence of domain D/A is relatively conserved within the various CSFV genotypes [29,31]. Several important conformational epitopes have been defined for the E2 protein [29,30,32,33]. In domain B/C, these include the highly conserved antigenic motif 753RYLASLHKKALPT765 [32], which is implicated in the production of neutralizing antibodies, and the motif 771LLFD774, which is essential for maintaining the structural integrity of conformational epitopes [29]. Residues D705, E713, D729 and K761 are responsible for the antigenic specificities of different genotypes [33]. Neutralizing epitopes are also present in domain D/A, and residue R845 is responsible for the antigenic specificity of different CSFV genotypes [30]. Another neutralizing conformational epitope with the antigenic determinant residue E902 is present in the *C*-terminal region [30]. In addition, two important linear epitopes are also present within E2, including the motif 772LFDGTNP778, which borders domains B/C and A [34,35], and the highly conserved CSFV-specific motif 829TAVSPTTLR837 in domain A [36,37]. Epitopes that are involved in virus neutralization represent the most important targets for antigenic evolution under selection pressure imposed by vaccines. Changes within these epitopes are associated with strong reductions in neutralizing titers against heterologous viruses [13].

The crystal structures of the E2 protein of BVDV can be divided into three [38] or four domains [39]. The E2 proteins of CSFV and BVDV share similar antigenic topologies [13]. Domain DA of BVDV corresponds to domain B/C of CSFV, and domain DB corresponds to domain D/A of CSFV [39]. According to another study, domains I and II of BVDV correspond to domains B/C and D/A of CSFV, respectively [38]. For BDV, however, little information is available regarding the epitopes and antigenic determinant residues of the E2 protein. A common epitope of CSFV and BDV has been mapped within the CSFV E2 domain B [40], wherein the residue L710 is essential for mAb recognition. A linear epitope 995YYEP998 is highly conserved among pestiviruses in the *C*-terminal end of E2 [41].

To understand the mechanism of the serological cross-reactivity among pestiviruses, it is important to identify the common or species-specific epitopes on E2. In the present study, we demonstrate the presence of a CSFV-specific conformational epitope and a CSFV and BDV common conformational epitope within the domain B/C of glycoprotein E2. These newly identified epitopes might be implicated in the early steps of CSFV infection and may facilitate the differential diagnosis of porcine pestivirus infections.

## 2. Materials and Methods

### 2.1. Cells and Viruses

Porcine kidney-15 (PK-15), sheep choroid plexus (SCP), sheep thymus (SFT-R) and Madin–Darby bovine kidney (MDBK) cells were maintained in minimum essential medium (MEM) supplemented with 10% fetal bovine serum (FBS), which tested free for pestivirus genomes and antibodies, and incubated at 37 °C in 5% CO_2_. The cell line SFT-R (CCLV-RIE 43) originated from the Collection of Cell Lines in Veterinary Medicine (CCLV, Friedrich-Loeffler-Institute, Island Riems, Greifswald, Germany) and the MDBK cell line was obtained from the American Type Culture Collection (Rockville, MD, USA). *Spodoptera frugiperda* (Sf9) cells were cultured at 27 °C in Grace’s Insect Medium, Supplemented (Thermo Fisher Scientific, Waltham, MA, USA) containing 10% FBS.

A panel of pestiviruses (including 21 CSFV strains, six BDV strains, two BVDV strains and pestivirus strain Aydin) representative of different pestivirus species and genotypes of CSFV and BDV (Table 1) were propagated at the EU and OIE Reference Laboratory for CSF, Institute of Virology, University of Veterinary Medicine Hannover, Germany (Table 1). The CSFV strains TD/96/TWN (designated TD/96 of genotype 2.1), 94.4/IL/94/TWN (designated 94.4 of genotype 3.4) and LPC/AHRI (designated LPC of genotype 1.1) [42,43,44] were propagated in PK-15 cells at the Animal Health Research Institute (AHRI), Taiwan. The CSFV strains Alfort/187 and UK2000/7.1 were propagated on PK15 cells and the BDV strain 137/4 was grown in SCP cells at the Animal and Plant Health Agency (APHA), the United Kingdom. Recombinant baculoviruses were grown and passaged in Sf9 cells at AHRI.

### 2.2. Antibodies Specific for Pestiviruses

Six mAbs, namely, 1C7A1, 3C1E12, WH220, WH304, WS381 and WS384, were used for epitope mapping. Two mAbs, WH303 and BVD/C16, were applied as control antibodies. The mAbs 1C7A1 and 3C1E12 were derived from the CSFV strain TD/96 by AHRI. The mAbs WH220, WH303, WH304, WS381 and WS384 were produced by APHA. The mAb WH220 was derived from the CSFV strain Baker/A of genotype 1.2 and the mAbs WH303 and WH304 were generated against the CSFV strain UK/86/2 of genotype 2.3. The mAbs WS381 and WS384 were derived from the BDV strain 87/6 of genotype 1.b [45,46]. The mAb BVD/C16 (Institute of Virology, University of Veterinary Medicine Hannover, Germany [47]) was generated against the BVDV strain NADL. This mAb was used as an infection control confirming that the cell layer was 70–90% infected by the corresponding viruses listed in Table 1. Polyclonal CSFV antiserum that was applied as positive control for the inhibition assay was collected from a pig immunized with the LPC vaccine.

### 2.3. Reactivity of a Panel of Pestiviruses to mAbs Specific for Glycoprotein E2

To investigate if the mAbs are specific to certain pestiviruses or pestivirus species, cells were infected with the corresponding viruses (Table 1) and seeded in 96-well plates to obtain an infection rate of 70–90% of the cell layer. Infections with all CSFV strains and the BDV strain Aveyron were performed on PK-15 cells, whereas for infections with the remaining BDV strains as well as with the pestivirus strain Aydin, SFT-R cells were applied. For infections with BVDV, MDBK cells were used. At 72 h post infection (hpi) the cells were fixed by heat treatment at 80 °C for 3 h (PK-15 cells) or 5 h (SFT-R and MDBK cells) and an indirect immune-peroxidase assay was performed. For this purpose, the fixed cell layer was rehydrated by adding phosphate-buffered saline supplement with 0.01% Tween20 (PBS-T). For antigen detection, the infected cells were incubated with the corresponding mAbs (dilution 1:50 in PBS-T) for 1 h at room temperature at 37 °C in combination with the species-specific secondary antibody (Polyclonal Rabbit Anti-Mouse Immunoglobulins/HRP, Agilent Dako; dilution 1:200 in PBS-T containing 4% horse serum). Thereafter, antigen detection was visualized by adding the chromogen substrate solution. After each incubation, the cell layer was subsequently washed three times with PBS-T. To confirm that the cell layer was 70–90% infected with the corresponding pestiviruses, the broad-reactive pestivirus NS3-specific mAb BVD/C16 was applied in addition to the E2-specific mAbs.

### 2.4. Construction of Various CSFV E2 Antigen Domains

Various domains of the E2 gene from the three CSFV strains TD/96, 94.4 and LPC, respectively, were amplified and cloned into the pMelBac A vector (Thermo Fisher Scientific) as previously described [29]. Domain B/C contained amino acid residues 690–779, B/C/D contained residues 690–806, C/D/A contained residues 717–869, D/A contained residues 770–869, B/C/D/A contained residues 690–869, and full-length CSFV-E2 constructs contained residues 690–1062 of the viral polyprotein (Table 2). The full-length E2 gene of BDV strain Moredun was also amplified and cloned into the pMelBac A vector.

### 2.5. Site-Directed Mutagenesis of CSFV and BDV E2

To replace the different amino acids on E2 of the CSFV strains TD/96, 94.4 and LPC, and the BDV Moredun strain, a series of single amino acids at positions 693, 709, 713, 725, 737 and 738 of CSFV and residues 709 and 713 of BDV were individually substituted, respectively (Table 3). Recombinant plasmids pMelBac A containing full-length E2 of CSFV strains TD/96, 94.4 and LPC, and the BDV Moredun strain were used as templates. All mutants were generated using the GeneArt^TM^ Site-Directed Mutagenesis System (Thermo Fisher Scientific) according to the manufacturer’s instructions. The resulting substitutions were verified by nucleotide sequencing.

### 2.6. Generation of Recombinant Baculoviruses

Recombinant plasmids pMelBac A (4 μg) were co-transfected with 0.5 μg of Bac-N-Blue^TM^ DNA (Thermo Fisher Scientific) into Sf9 cells by Cellfectin^®^ II Reagent (Thermo Fisher Scientific). The recombinant baculoviruses were harvested after incubation at 27 °C for 72 h. The purified and high-titer recombinant baculoviruses were prepared with Sf9 cells according to the manufacturer’s instructions.

### 2.7. E2 Proteins Expression and Purification

E2 proteins were collected from the medium of SF9 cells infected with recombinant baculoviruses and then purified with HisPur Cobalt Resin (Thermo Fisher Scientific) following the manufacturer’s instructions. The purified proteins were subsequently concentrated with 10 K (10,000 NMWL) Amicon^®^ Ultra-15 Centrifugal Filters (Merck Millpore, Darmstadt, Germany) and Halt^TM^ EDTA-free protease inhibitor cocktail was added (Thermo Fisher Scientific). The concentrations of purified protein were determined using the Pierce^TM^ BCA protein assay kit (Thermo Fisher Scientific).

### 2.8. Virus Cross-Neutralization Test of mAbs Specific for Glycoprotein E2

Two-fold serially diluted mAbs specific for E2 were mixed with equal volumes of 100 TCID_50_ of the CSFV strains TD/96, 94.4, LPC, Alfort/187 and UK2000/7.1 or the BDV strain 137/4, incubated at 37 °C for 1 h, and subsequently transferred to PK-15 (applied for CSFV) or SCP (applied for BDV) cells in 96-well plates. The starting dilution of each mAb (at a concentration of 1mg/mL) was 1:4, 1:5 or 1:8. After 72 hpi, the cells were fixed and stained for the presence of pestivirus antigen by indirect fluorescent assay (IFA; Section 2.12) (TD/96, 94.4 and LPC) or after 120 hpi by indirect immune-peroxidase assay (Alfort/187, UK2000/7.1 and 137/4). The neutralizing titer is the log2 of the antibody dilution factor (reciprocal of dilution) when 50% of the wells are protected from infection.

### 2.9. Inhibition of CSFV Binding to Cells by Full-Length E2 and Domain B/C

Confluent monolayers of PK-15 cells, grown in 24-well plates, were pre-cooled at 4 °C for 1 h. This condition prevents receptor-mediated endocytosis while allowing virus attachment and binding to the receptor. Thereafter, the cells were washed three times with ice-cold PBS and then incubated with the purified full-length E2 protein of the CSFV strain TD/96 or with the E2 protein domain B/C using 20, 50, or 100 μg per 24-well at 4 °C for 1 h. As a blank control, 100 µg of bovine serum albumin (BSA) was applied. Then, the TD/96 strain, at a multiplicity of infection (MOI) of 1, was added to infect the cells. After 1 h of incubation at 4 °C, unbound viruses were removed, the cells were washed three times with ice-cold PBS, and fresh PBS was added. The viruses were harvested by three cycles of freeze and thaw. The infectious cell culture supernatant was clarified by centrifugation at 3000× *g* for 10 min to remove cell debris and virus titration was performed. The inhibition percentage of virus binding to cells by E2 proteins was calculated according to the formula: 100 × [(1 − (*e*/*c*)]. Here, *c* and *e* represent the viral titer after treatment without or with E2 proteins, respectively.

### 2.10. Inhibition of CSFV Binding to Cells by mAbs Specific for Glycoprotein E2 and Polyclonal CSFV Antiserum

The TD/96 strain at MOI 1 was added to mAbs specific for E2 at 10 μg each or to polyclonal CSFV swine antiserum at 4 °C for 1 h, respectively. Mouse and swine serum seronegative for CSFV were used as a blank control for mAbs or polyclonal swine serum, respectively. Confluent monolayers of PK-15 cells, grown in 24-well plates, were pre-cooled at 4 °C for 1 h before they were washed three times with ice-cold PBS. The washed cells were incubated with the virus/antibody mixture and incubated at 4 °C for 1 h. To remove unbound viruses, the cells were washed with ice-cold PBS three times and fresh PBS was added. The viruses were harvested by three cycles of freeze–thawing. The supernatant was clarified by centrifugation at 3000× *g* for 10 min to remove cell debris and virus titration was performed. The inhibition percentage of virus binding to cells by antibodies was calculated as described above. Here, *c* and *e* represent the viral titer after treatment without or with antibodies, respectively.

### 2.11. Virus Titration

Ten-fold serially diluted CSFV harvested from inhibition assay (Section 2.9 and Section 2.10) were added into eight wells, each of 96-well plates, and seeded with PK-15 cells. Infection of cells was monitored by IFA (Section 2.12) at 72 hpi. Virus titers were calculated as TCID_50_ using the Reed–Muench method [48].

### 2.12. Indirect Fluorescent Assay (IFA)

To detect the expression of recombinant E2 proteins by anti-E2 mAbs, Sf9 cells in 96-well microtiter plates were infected with recombinant baculoviruses at a MOI of 10 and incubated for 4 days to achieve high-level expression. The inoculated cells were fixed with 10% formaldehyde at room temperature for 10 min and washed three times with PBS. Each anti-E2 mAb was diluted 1:100 in PBS and 50 μL was added per well. The cells were then incubated at 37 °C for 1 h and washed three times with PBS. Fluorescein isothiocyanate-conjugated goat anti-mouse IgG (Jackson ImmunoResearch Laboratories, West Grove, PA, USA), diluted 1:100 in PBS, was added, and the cells were incubated at 37 °C for 1 h and then washed three times with PBS. Fluorescence of the stained cells was observed under a fluorescence microscope (Olympus Imaging America, Center Valley, PA, USA).

Moreover, IFA was used for CSFV antigen detection in combination with virus titration (Section 2.11) and virus cross neutralization (Section 2.8). The mAb WH303, diluted 1:100 in PBS, was used for the detection of CSFV.

### 2.13. Statistical Analysis

For the inhibition of CSFV binding to porcine cells (Section 2.9 and Section 2.10), differences in the values among various groups were analyzed using one-way analysis of variance (ANOVA) combined with the Duncan’s multiple range test. The analysis was carried out in Statistical Analysis System (SAS) Enterprise Guide 7.1 (SAS Institute Inc., Cary, NC, USA). Mean differences were considered significant when the *p*-value was <0.05.

### 2.14. Alignment of the Amino Acid Sequences of Pestivirus Glycoprotein E2

The E2 amino acid sequences comprising residues 690 to 738 (representing the domain B/C of the CSFV E2 protein) of the pestiviruses that were analyzed in the current study were obtained from the National Center for Biotechnology Information (NCBI) or from the CSF database of the EU and OIE Reference Laboratory for CSF [49]. Protein alignment of these sequences was generated by using the Clustal W method of MegAlign-expert software (DNASTAR, Madison, WI, USA).

### 2.15. The 3D Structure Modeling of CSFV E2

The 3D structure of E2 of the CSFV strain TD/96 was modeled using the I-TASSER server (https://zhanglab.ccmb.med.umich.edu/I-TASSER/, accessed on 12 June 2020). The BVDV E2 glycoprotein (protein data bank code: 2YQ2) was used as a template [39]. The locations of the identified conformational epitopes on the predicted model of the E2 protein of CSFV were revealed by the PyMOL software (https://www.pymol.org, accessed on 15 August 2020).

## 3. Results

### 3.1. Reactivity of a Panel of Pestiviruses with mAbs Specific for Glycoprotein E2

The different binding patterns of E2-specific mAbs to a panel of pestiviruses (including representative strains of CSFV, BDV, BVDV and Aydin pestivirus) are summarized in Table 1. The mAbs 1C7A1, 3C1E12, WH220 and WH304 were derived from CSFV, and the mAbs WS381 and WS384 were generated against BDV. The mAbs 1C7A1, WH220 and WH304 only recognized CSFV strains, whereas the mAbs 3C1E12, WS381 and WS384 showed cross reactivity among pestiviruses.

The mAb 1C7A1 only recognized CSFV genotypes 2.1, 2.2 and two out of six CSFV strains of genotype 2.3. The mAb 3C1E12 clearly detected all CSFV and BDV genotypes, except for the CSFV genotype 1.1 strains, which reacted weakly, with only 2–4 cells staining positive, and also resulted in negative staining with CSFV genotype 3.1. The mAbs 1C7A1 and 3C1E12 were generated against the same CSFV strain TD/96 (genotype 2.1). However, the mAb 3C1E12 detected both CSFV and BDV, whereas mAb 1C7A1 did not recognize BDV.

The mAb WH220 could only recognize CSFV but did not detect genotypes 2.3, 3.1, and reacted weakly with genotype 3.4, with only 2–4 cells staining positive. The mAb WH304 represents the only antibody of the current study that is CSFV specific independently of the CSFV genotype. As the mAbs WS381 and WS384 were generated against BDV, both antibodies detected all tested BDV strains. With regard to CSFV, the mAb WS384 showed broad reactivity and detected also the pestivirus isolate Aydin/04-TR and the BVDV isolate CS8644. In comparison to this, the mAb WS381 only well recognized some CSFV genotype 2 strains. The mAb BVD/C16 recognized all pestiviruses and served as a control confirming that the cell layer was 70–90% infected with the corresponding pestiviruses.

**Table 1:** Analysis of a panel of pestiviruses with E2-specific mAbs ^a^.

Viruses			mAbs						
			CSFV ^c^				BDV		BVDV
Virus	Strain	Genotype	1C7A1	3C1E12	WH220	WH304	WS381	WS384	BVD/C16
CSFV	Alfort/187 (CSF0902 ^b^)	1.1	−	(±)	+	+	−	−	+
	Riems (CSF0940)	1.1	−	(±)	±	+	±	±	+
	Brescia (CSF0929)	1.2	−	+	+	+	−	−	+
	Baker A (CSF0932)	1.2	−	+	±	+	±	+	+
	VRI 4167 (CSF0306)	1.3	−	+	+	+	±	+	+
	Guatemala HC (CSF0650)	1.3	−	+	+	+	−	±	+
	39/Margarita (CSF0705)	1.4	−	+	±	+	±	±	+
	Pinar del Rio (CSF1058)	1.4	−	±	±	+	±	±	+
	V1240/97 (CSF0277)	2.1	+	+	−	+	+	+	+
	Panevezys (CSF1048)	2.1	+	+	±	+	±	+	+
	TD/96/TWN (CSF1077)	2.1	+	+	+	+	+	+	+
	Parma (CSF0573)	2.2	+	+	±	+	−	+	+
	Bergen (CSF0906)	2.2	+	+	+	+	+	+	+
	BG/Jambul (CSF0864)	2.3	+	+	−	+	±	+	+
	Alfort/Tuebingen (CSF0904)	2.3	+	+	−	+	±	+	+
	Diepholz I (CSF0104)	2.3	−	+	−	+	±	+	+
	VI 3837/38 (CSF0634)	2.3	−	+	−	+	+	+	+
	M7 19928/60 (CSF1027)	2.3	−	+	−	+	−	+	+
	Roesrath (CSF1045)	2.3	−	+	−	+	±	+	+
	Congenital Tremor (CSF0410)	3.1	−	−	−	+	−	−	+
	Kanagawa (CSF0309)	3.4	−	+	(±)	+	−	+	+
BDV	Moredun	1.a	−	+	−	−	+	+	+
	137/4	1.b	−	+	−	−	+	+	+
	Reindeer 1	2	−	+	−	−	+	+	+
	Gifhorn	3	−	+	−	−	+	+	+
	Chamois	4	−	+	−	−	+	+	+
	Aveyron	5	−	+	−	−	+	+	+
Aydin	Aydin/04-TR		−	−	−	−	−	+	+
BVDV-1	NADL		−	−	−	−	−	−	+
BVDV-2	CS8644		−	−	−	−	−	+	+

^a^ Results were interpreted as positive (+), weak (±), only 2–4 cells staining positive ((±)) or negative (−). ^b^ Numbers were the ID of the EURL Classical Swine Fever Virus Database. ^c^ Derived from CSFV, BDV or BVDV.

### 3.2. Reactivity of Different CSFV E2 Antigen Domains with E2-Specific mAbs

The reactivity of E2-specific mAbs to various CSFV E2 antigen domains is summarized in Table 2. The domain B/C was present in four tested constructs (B/C, B/C/D, B/C/D/A and full-length). The reactivity of each mAb with these four constructs (in dependent of the virus strains that was used for the generation of the construct) was comparable. The mAbs 1C7A1 and WS381 only recognized the expressed proteins of the CSFV strain TD/96 (genotype 2.1) and showed no reactivity against the expressed proteins of the 94.4 (genotype 3.4) and LPC (genotype 1.1) strains. The mAbs 3C1E12 and WS384, despite being respectively derived from CSFV and BDV, had identical reactivity patterns. They recognized the expressed proteins of the CSFV strains TD/96 and 94.4 but did not recognize the proteins of the LPC strain. The mAb WH220 recognized the expressed proteins of the CSFV strains TD/96 and LPC, but not the 94.4 strain. The mAb WH304 detected expressed proteins derived from all three CSFV strains. These findings are generally consistent with the results obtained from the reactivity patterns detected by analyzing the mAbs with different CSFV genotypes (Table 1).

If the domain B/C was missing (constructs C/D/A and D/A), none of the antibodies were able to bind to these domains, confirming that the six mAbs target the domain B/C. Protein expression was confirmed by the control mAb WH303, which targets the 829TAVSPTTLR837 motif within domain A [36].

**Table 2:** Antigenic analysis of different domains of CSFV E2 protein by IFA with E2-specific mAbs ^a^.

Domain	Strain ^b^	mAbs against E2						
		Domain B/C						Domain A
		CSFV ^c^				BDV		CSFV
		1C7A1	3C1E12	WH220	WH304	WS381	WS384	WH303
B/C (aa 690–779)	TD/96	+	+	+	+	+	+	−
	94.4	−	+	−	+	−	+	−
	LPC	−	−	+	+	−	−	−
B/C/D (aa 690–806)	TD/96	+	+	+	+	+	+	−
	94.4	−	+	−	+	−	+	−
	LPC	−	−	+	+	−	−	−
C/D/A (aa 717–869)	TD/96	−	−	−	−	−	−	+
	94.4	−	−	−	−	−	−	+
	LPC	−	−	−	−	−	−	+
D/A (aa 770–869)	TD/96	−	−	−	−	−	−	+
	94.4	−	−	−	−	−	−	+
	LPC	−	−	−	−	−	−	+
B/C/D/A (aa 690–869)	TD/96	+	+	+	+	+	+	+
	94.4	−	+	−	+	−	+	+
	LPC	−	−	+	+	−	−	+
Full-length (aa 690–1062)	TD/96	+	+	+	+	+	+	+
	94.4	−	+	−	+	−	+	+
	LPC	−	−	+	+	−	−	+

^a^ Results were interpreted as positive (+) (grey highlights) or negative (−). ^b^ TD/96, 94.4 and LPC indicated CSFV strains TD/96/TWN (genotype 2.1), 94.4/IL/94/TWN (genotype 3.4) and LPC/AHRI (genotype 1.1), respectively. ^c^ Derived from CSFV or BDV.

### 3.3. Virus Cross-Neutralization Titers of mAbs Specific for Glycoprotein E2

To find out whether the E2 specific mAbs have a virus neutralization capacity, the neutralizing potential of the E2 mAbs toward different CSFV genotypes and BDV was determined by cross-neutralization assay (Table 3). The mAbs 1C7A1 and 3C1E12 binding to domain B/C neutralized the CSFV genotype 2.1 strain TD/96, but not the CSFV strains LPC (genotype 1.1), Alfort/187 (genotype 1.1) and 94.4 (genotype 3.4). The mAb 1C7A1 showed a high neutralization titer (ND_50_ > 512) against the TD/96 strain but did not neutralize other CSFV or BDV strains tested. The neutralization titers of mAb 3C1E12 were ND_50_ = 64 and ND_50_ = 640 against CSFV genotype 2.1 strains TD/96 and UK2000/7.1, respectively, consistent with its restriction to the detection of CSFV genotype 2 strains (Table 1). The higher neutralization titer of ND_50_ > 512 obtained for mAb 1C7A1 to the TD/96 strain, compared to the low titer of ND_50_ = 64 by mAb 3C1E12, correlated with the higher specificity of mAb 1C7A1 to TD/96, from which both mAbs derived. In addition, mAb 3C1E12 showed a high neutralization titer (ND_50_ = 1280) against the BDV strain 137/4, consistent with its broad reactivity detected for BDV (Table 1). The mAb WH220 did not neutralize any of the strains tested, whereas the mAb WH304 produced variable results. Evidence of low neutralizing ability was observed against CSFV strains Alfort/187, 94.4 and potentially against the BDV 137/4 isolate, indicating that WH304 may only have borderline neutralizing ability. The mAb WS381 neutralized the BDV strain 137/4, whereas the mAb WS384 only showed low neutralizing activity against this BDV strain in two replicate assays. The mAb WH303 against domain A neutralized three CSFV strains belonging to various genotypes, in which the 94.4 strain was neutralized the most (ND_50_ = 256), followed by the LPC strain (ND_50_ = 64) and the TD/96 strain (ND_50_ = 8). The neutralizing ability of mAb WH303 against the other CSFV strains and against BDV was not tested.

**Table 3:** Virus cross-neutralization titers of E2-specific mAbs.

mAb.	Derived from (genotype)	Domain	Neutralization Titer					
			LPC ^a^ (1.1)	Alfort/187 ^b^ (1.1)	TD/96 ^a^ (2.1)	UK2000/7.1 ^b^ (2.1)	94.4 ^a^ (3.4)	BDV 137/4 ^b^ (1.b)
1C7A1	CSFV TD/96 (2.1)	B/C	<4	<5	>512	<5	<4	<5
3C1E12	CSFV TD/96 (2.1)	B/C	<4	<5	64	640	<4	1280
WH220	CSFV Baker A (1.2)	B/C	<8	<5	<8	<5	<8	<5
WH304	CSFV UK/86/2 (2.3)	B/C	<8	14 ^c^	<8	<5	16	6 ^c^
WS381	BDV 87/6 (1.b)	B/C	<8	<5	<8	≤7	<8	376
WS384	BDV 87/6 (1.b)	B/C	<8	<5	<8	≤5	<8	14 ^c^
WH303	CSFV UK/86/2 (2.3)	A	64	n.t.	8	n.t.	256	n.t.

n.t. = not tested. ^a^ TD/96, 94.4 and LPC indicated CSFV TD/96/TWN, 94.4/IL/94/TWN and LPC/AHRI strains, respectively. These strains were tested at AHRI. ^b^ The CSFV strains Alfort/187, UK2000/7.1 and the BDV strain 137/4 were tested at APHA. ^c^ Variable results were obtained with mAbs WH304 and WS384. Results are the mean values of two or three independent assays.

### 3.4. Inhibition of CSFV Binding to Cells by Full-Length E2 and Domain B/C

**Figure 1:**
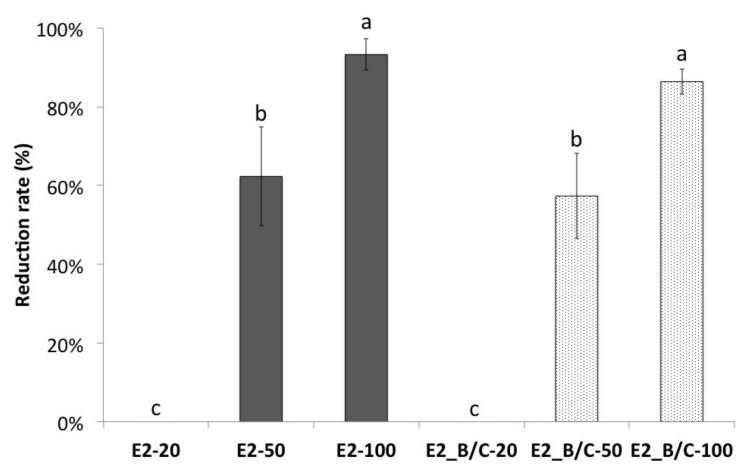
Inhibition of CSFV binding to PK15 cells by recombinant expressed full-length or partial E2 proteins. For this purpose, the full-length E2 protein and domain B/C (E2_B/C) were used. The numbers within the names of the E2 constructs refer to the protein amounts (µg) per 24-well. The data represent the mean and standard deviation of three independent experiments. Values with different superscript letters, a–c, indicate a statistically significant difference (*p* < 0.05) from each other. The superscript letter “a” indicates the highest reduction of binding and “c” indicates unchanged binding to cells No significant differences exist between values containing the same letter.

To study the importance of the E2 domain B/C in virus binding to porcine cells, the recombinant expressed E2 domain B/C as well as the full-length E2 protein of CSFV strain TD/96 were used to block cell binding. Both E2 constructs inhibited the binding of the homologous CSFV strain TD/96 to the porcine cells in a dose-dependent manner (Figure 1). Protein of 20 µg per 24-well had no significant effect on binding to cells. However, an increase of the amount to 50 µg reduced the infectivity to 62% (full-length E2 protein) and 57% (domain B/C of the E2), respectively. In comparison to this, 93% (full-length E2) and 86% (domain B/C) inhibition of virus binding was detected when 100 µg of each protein was applied, respectively. This inhibition was significantly higher compared to the reduction caused by 50 μg (*p* < 0.05). These results strongly suggested that the domain B/C of the CSFV E2 is involved in the binding of CSFV to porcine cells.

### 3.5. Inhibition of CSFV Binding to Porcine PK15 Cells by E2-Specific mAbs and Polyclonal CSFV Antiserum

**Figure 2:**
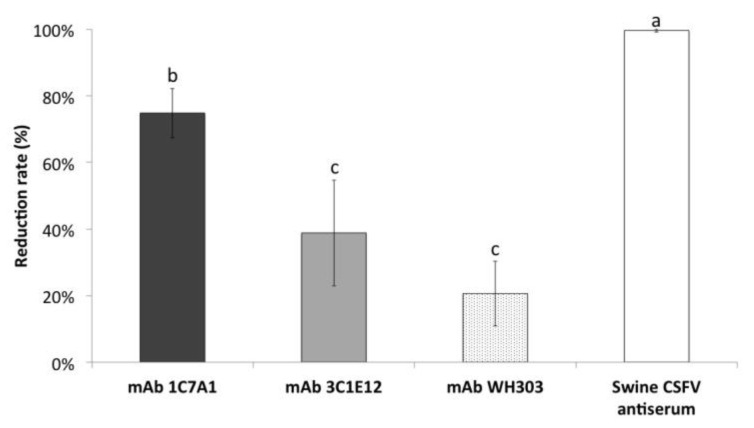
Inhibition of CSFV binding to porcine cells by three mAbs specific for glycoprotein E2 and polyclonal CSFV antiserum. An equal amount of each mAb was used (10 µg). The data represent the mean and standard deviation of three independent experiments. Values with different superscript letters, a–c, indicate a statistically significant difference (*p* < 0.05) from each other. The superscript letter “a” indicates the highest and “c” indicates the lowest reduction of binding to cells among the compared groups. No significant differences exist between values containing the same letter.

In addition, the inhibition of CSFV binding to cells was analyzed by using two mAbs, which recognize the domain B/C and were used for pre-incubation with the virus before the cells were infected. As a control, a polyclonal CSFV antiserum was applied. The polyclonal CSFV antiserum completely inhibited the binding of the CSFV strain TD/96 to porcine cells. In comparison to this, the mAb 1C7A1 led to a reduction of the virus binding capacity of up to 75%. This was significantly higher (*p* < 0.05) compared to the reductions caused by mAb 3C1E12 (39% reduction) and by the control antibody WH303 (21% reduction), which targets the A domain (Figure 2). The reduction rates of these mAbs correlated with their neutralization titers against the TD/96 strain, in which mAb 1C7A1 had the highest titer (ND_50_ > 512), followed by mAb 3C1E12 (ND_50_ = 64) and mAb WH303 (ND_50_ = 8) (Table 3). Taken together, the higher blocking activity of mAb 1C7A1, compared to mAb 3C1E12, correlates with the higher specificity and with the higher neutralizing activity against CSFV strain TD/96, from which both mAbs derived.

### 3.6. Alignment of the Amino Acid Sequences of Pestiviruses Glycoprotein E2

**Figure 3:**
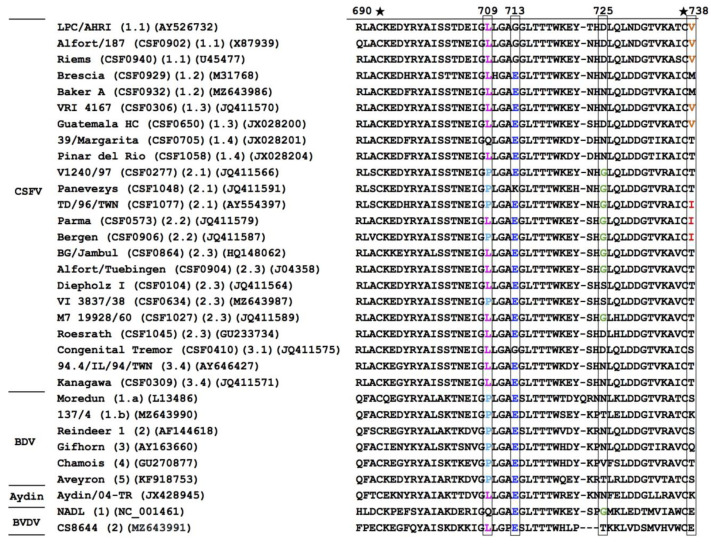
Alignment of the amino acid sequences of residues 690–738 of pestivirus glycoprotein E2. The positions of amino acid residues are numbered based on those of CSFV strain TD/96/TWN. Sequences are clustered based on their allocation to genotypes as in Table 1. For each virus strain the genotype and the accession number are indicated. The key residues which might be responsible for the reactivity patterns of the mAbs characterized in this study are boxed and highlighted with different colors. The residues P709, L709, E713, G725, V738 and I738 are highlighted with light blue, pink, blue, green, orange and red, respectively. Cysteine residues are indicated by an asterisk above the alignment.

To identify species or genotype specific residues that may be implicated in the recognition by the different mAbs, a protein sequence alignment was performed using the amino acid residues of domain B/C of the E2 protein (residues 690 and 738) of the pestiviruses that were tested in the current study (Figure 3). Cysteines at the positions 693 and 737 of CSFV are conserved among pestiviruses, as shown in a previous study [4]. Residue P709 is conserved among BDV strains and is present in some CSFV genotype 2 protein sequences. Among the other pestiviruses, amino acid L709 is conserved, except for one strain belonging to the CSFV genotype 1.4 and the BVDV strain NADL. Residue E713 is conserved among most CSFV strains and the other pestiviruses, except for genotype 1.1 strains, one strain of genotype 2.1 and one strain of genotype 3.1 of CSFV. Residue G725 is specific for CSFV genotype 2.1 and 2.2 strains and is present in some CSFV genotype 2.3 strains. Residue V738 is specific for CSFV genotype 1.1 and 1.3 strains, while I738 is specific for the CSFV genotype 2.1 strain TD/96 and genotype 2.2 strains. On the basis of this comparative sequence analysis, it was hypothesized that these amino acid differences might be responsible for the observed differences in antibody reactivity with the different CSFV strains, BDV strains or other pestiviruses (Table 1), and that these residues are likely candidates of antigen-specific residues. To investigate this hypothesis, these residues were modified by site-directed mutagenesis and subjected to further analysis.

### 3.7. Identification of Critical Residues of E2 for mAbs Binding

To identify residues critical for binding of the different mAbs to CSFV or BDV E2 proteins, site-directed mutagenesis was performed (Table 4). Intramolecular disulfide bridges are responsible for the correct folding of the proteins and thus important for the formation of conformational epitopes. Substitutions of cysteines at the positions 693 and 737 of the domain B/C abrogated the binding of all mAbs, which target domain B/C (Table 4). Residue G725 was critical for epitope detection by mAb 1C7A1, because mutation of G725D of TD/96 E2 abolished antibody binding, while mutant D725G in the E2 protein of the CSFV vaccine strain LPC gained recognition by the same mAb. Mutagenesis of other residues did not affect the binding of mAb 1C7A1, as compared to the non-mutated E2. The critical residue of the epitope recognized by mAb WH220 was I738/V738.

Residue E713 was important for the epitope detection of both mAb 3C1E12 and WS384, again highlighting their rather similar reactivity (see Table 2). Amino acid changes at this location resulted in similar alterations in antibody binding. In addition to residue E713, residue P709 was implicated in binding of the mAb WS384, but this location did not appear to contribute to 3C1E12 reactivity. P709 was also an important amino acid involved in the binding of mAb WS381. In addition, residue L709 was critical for the binding of the mAb WH304 to the epitope of the CSFV strain LPC and BDV strain Moredun. All the CSFV mutated E2 proteins were recognized by mAb WH303, which served as a control for protein expression. The alteration of these four residues resulted in a binding pattern that corresponded closely with the reactivity pattern detected by analyzing the mAbs with the tested pestiviruses (Table 1).

### 3.8. The 3D Structure Modeling of CSFV E2

**Figure 4:**
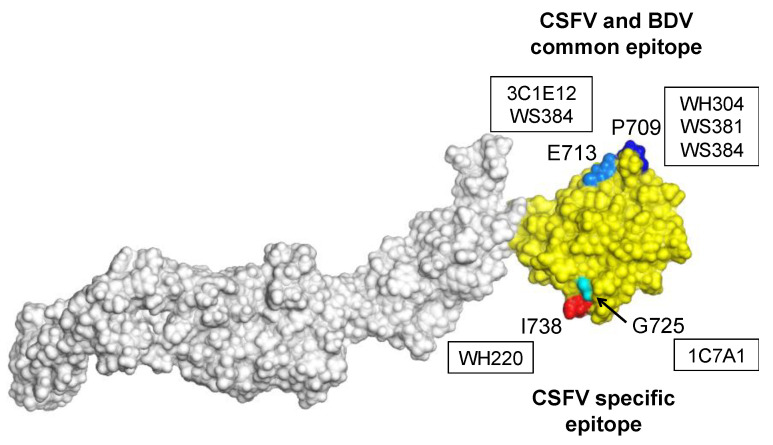
The 3D structure modeling of E2 of CSFV strain TD/96. The domain B/C is colored yellow. The residues P709, E713, G725 and I738 recognized by mAbs are highlighted with dark blue, light blue, light green and red, respectively. The mAbs recognizing the highlighted amino acid residues are indicated in boxes.

The 3D structure of E2 of the CSFV strain TD/96 was modeled using the BVDV E2 glycoprotein (protein data bank code: 2YQ2) [39] as the template and is shown in Figure 4. The PyMOL visualization revealed that the residues P709, E713, G725 and I738 recognized by mAbs characterized in the present study are exposed on the surface of domain B/C. The residues G725 and I738 specific for CSFV are located near each other, while the residues P709 and E713, that are conserved and shared by both CSFV and BDV, are also located near each other.

## 4. Discussion

Members of the genus *Pestivirus*, mainly CSFV, BDV and BVDV, can infect swine, eliciting cross-reactive antibodies that can cause problems in the serological diagnosis of CSF [5]. In addition, Aydin pestivirus and other newly identified ruminant pestiviruses are genetically and antigenically closely related to both CSFV and BDV [8,9]. The close antigenic relationship to CSFV has been observed in the strong cross-reactivity of sera from sheep and goats in CSFV-specific ELISAs and neutralization assays [8,9,10,11], increasing the need to be able to distinguish BDV and other ruminant pestiviruses from CSF. For serological differentiation, it is important to identify common epitopes of pestiviruses and specific epitopes of CSFV; this might also contribute to the development of improved DIVA vaccines.

Pestiviruses are structurally and antigenically related [4]. A linear epitope in the *C*-terminal part of E2 is highly conserved among pestiviruses [41], suggesting a similar antigenic structure of E2 among pestiviruses. The crystal structures of the BVDV E2 protein revealed that CSFV and BVDV E2 proteins possess similar topologies [13,38,39]. However, a study by van Rijn [40] showed that none of the mAbs derived against E2 of BVDV-I and BVDV-II recognized E2 of CSFV, whereas a mAb derived against E2 of BDV detected the E2 protein of CSFV and partly neutralized CSFV. The corresponding common epitope of CSFV and BDV has been identified within the antigenic domain B of the CSFV E2 protein [40]. Interestingly, the present study identified a common conformational epitope of CSFV and BDV, located in the E2 antigenic domain B/C. Binding and neutralization assays as well as 3D structural modeling indicate that this epitope is exposed on the surface of virions. Two mAbs (3C1E12 and WS381) detected CSFV and BDV, but not Aydin or BVDV, suggesting that CSFV shares a higher degree of antigenic similarity in this region with BDV than with Aydin/04/TR pestivirus or BVDV. However, a study by Postel et al. [8] showed by cross neutralization assays that Aydin pestivirus is antigenically more closely related to CSFV than to BDV. A possible explanation is that CSFV and Aydin might have common epitopes other than the epitope of CSFV E2 and BDV E2 or of other proteins, which could also induce neutralizing antibody responses.

The cysteine residues C693 and C737 are conserved among CSFV, BDV, Aydin pestivirus and BVDV. Mutation of either C693 or C737 abrogated the reactivity of the investigated mAbs with domain B/C, indicating that the disulfide bond between C693 and C737 is essential for correct folding of domain B/C and structural integrity of conformational epitope recognition, consistent with previous findings [28,29]. However, compared to CSFV, other pestiviruses, such as BDV, Aydin pestivirus and BVDV contain two additional cysteine residues at position 748 of domain B/C and position 794 of domain D/A in the N-terminal half of E2 [4]. These cysteines could form an additional disulfide bond that can potentially change the antigenic structure of E2, as indicated by the crystal structure of BVDV E2 [38,39]. However, this study showed a similar antigenic structure of CSFV and BDV, suggesting that residues C748 and C794 might not cause a significant change in the antigenic structure of E2. Further analyses of E2 proteins with mutations of these cysteines are required to confirm this assumption.

The amino acids important for the binding of the six mAbs to the domain B/C were further examined by site-directed mutagenesis. A single amino acid replacement within the domain B/C of the full-length E2 protein of the opposite strain (i.e., the field TD/96 and 94.4 viruses versus LPC vaccine) could totally reverse the binding pattern, based on the premise that the structural integrity of the conformational epitope is not disturbed; this observation is consistent with previous studies [30,33]. Four residues were defined to be important for antigen–antibody interactions: residues at the positions 709, 713, 725 and 738. The substitutions at P709 → G/L, E713 → G, G725 → D, and I/V738 → T caused a dramatic effect on the topography of antigens and thus reversed the antibody binding. The alteration of these residues resulted in a binding pattern that corresponded closely with the reactivity pattern detected by analyzing the mAbs with the tested pestiviruses. However, according to the protein alignment, there are some exceptions. For example, the residue G725 is important for binding of mAb 1C7A1, but two pestivirus strains (CSF1027 and BVDV NADL) with residue G725 were not detected by this mAb. Another example is that the residue E713 is important for binding of mAb 3C1E12, but despite the presence of residue E713, the pestivirus strain Aydin and BVDV were not detected by this mAb. A reason for this might be that other amino acid differences are involved, which may lead to structural changes of the protein, or possibly that these amino acid differences are part of the epitope and reduce the binding of the mAb to the epitope.

The amino acids important for antibody binding to the common conformational epitope of CSFV and BDV at domain B/C were identified as L710 [40], P709/L709 and E713. The three identified residues are located near each other (Figure 4) within the domain B/C of E2 and might form the same conformational epitope, as residues P709 and E713 of CSFV and BDV are both critical for the binding of mAb WS384. Interestingly, residue L709 is critical for binding of the epitope of the CSFV strain LPC and the BDV strain Moredun to mAb WH304. However, five CSFV strains with residue P709 are also recognized by mAb WH304 (Table 1). In addition, residue P709 is a critical residue of CSFV and BDV recognized by mAbs WS381 and WS384. Proline has a unique cyclic structure compared to other amino acids and plays significant roles in the folding of proteins, so changes at this residue are likely to have significant impacts on the antigenic structures of the E2 of different CSFV and BDV strains.

Another interesting finding is that mAbs 3C1E12, WS381 and WS384 recognized CSFV and BDV, but could not or could only weakly detect the CSFV genotype 1.1. Residue E713 of CSFV and BDV is critical for binding of mAbs 3C1E12 and WS384, while residue 713 of CSFV genotype 1.1 strains is glycine. Previous studies demonstrated that residue E713 is a major determinant of antigenic variation of CSFV field strains. The G713E substitution enhanced the recognition of recombinant vaccine strains and mutated E2 protein belonging to genotype 1.1 by mAbs [33,50] or swine serum against CSFV genotype 2.1 [51]. This present study is the first report demonstrating that residue E713 represents an important antigenic determinant residue of a common conformational epitope shared by both CSFV and BDV.

Cellular attachment and entry are the first steps of virus infection to host cells. Glycoproteins E1 and E2 of CSFV form an E1–E2 heterodimer, located in the viral envelope that mediates initial viral binding to the cell and entry [15]. To determine whether CSFV binding to cells would be inhibited by E2 proteins or antibodies, PK-15 cells were incubated with a virus at 4 °C, a temperature at which virus binding can take place but endocytosis is prevented. Virus binding to PK-15 cells was significantly reduced in a dose-dependent manner when cells were pre-incubated with E2 protein, consistent with previous studies [14,15]. Our results further demonstrated that a partial E2 protein encompassing domain B/C only could also efficiently inhibit CSFV infection. This indicated the strong involvement of domain B/C in virus binding to cells. In addition, virus binding to PK-15 cells was significantly reduced when CSFV was pre-incubated with E2-specific mAbs binding to domain B/C or polyclonal CSFV anti-serum. Despite the possibility of a steric block due to the binding of large molecules, e.g., antibodies, the different reduction rates of the investigated mAbs (Figure 2), correlating with their neutralization titers (Table 3), support the assumption that the conformational epitopes recognized by the mAbs are located within a receptor-binding domain (RBD) of CSFV E2 protein. Further experiments including generation of recombinant CSF viruses expressing E2 proteins with mutations of the corresponding residues are necessary to confirm that these epitopes are crucially involved in virus binding to the host cells. The 3D structure modeling of CSFV E2 (Figure 4) showed that the identified residues are located on the surface of domain B/C. One epitope contains the residues G725 and V738/I738 specific for CSFV, and the other contains the residues P709/L709 and E713 conserved in both CSFV and BDV.

The mAb WH303 binding to domain A of CSFV E2 showed virus neutralizing capacity (Table 3 and Figure 2). Domain D/A of CSFV E2 refers to domain II of BVDV E2, and a stretch of amino acids in domain II of BVDV E2 has been identified as a possible receptor ligand [38]. A recent study also identified an exposed β-hairpin motif in domain II of BVDV E2 that mediates receptor binding [52]. Whether a similar epitope of domain D/A of CSFV is located in RBD is under investigation.

The amino acid sequences and protein structure of glycoprotein E2 are involved in determining the host tropism of different pestiviruses, as the inhibition of binding to permissive and non-permissive cells by E2 proteins is higher when using E2 derived from the homologous pestivirus [18]. According to a recent study, the residue at position 739 of BDV E2 (corresponding to the residue at position 738 of CSFV E2) is required for viral entry into cells and determines the host tropism for sheep and pig cells, as the substitution of residue Q739 of BDV E2 to either of the positively charged residues R or K was crucial for BDV to adapt to pig cells [19]. It was assumed that the amino acid substitutions to positively charged amino acids might affect BDV attachment and entry and thus enhance virus replication in pig cells [19]. However, as the residues at the same position of most CSFV strains are V, I, or T (Figure 3), which are hydrophobic or polar but not positively charged amino acids, the role of the residues at positions 738 in CSFV and 739 in BDV in virus attachment, cell entry, and host adaption to pigs needs further investigation. Interestingly, the residue at position 738 is important for mAb WH220 binding, but this antibody did not neutralize the CSFV strains or the one BDV strain tested.

Since E2 protein of CSFV plays an important role in inducing neutralizing antibody responses, it is hypothesized that mutations arise as a result of immune selection. Positive selective pressure has acted on CSFV E2 glycoprotein [53,54,55,56,57,58], including the residues at positions 709 [54,55], 725 [56] and 738 [55,56,57,58] of domain B/C, which are the main targets for antigenic evolution under selection pressure imposed by vaccine mediated immunity. Previous studies showed that E2-specific mAbs had higher neutralization efficacy against homologous CSFV strains [51,59], which is consistent with our results (Table 3). This suggests that a minor alteration of antigenic epitopes at domain B/C of E2 of CSFV strains can be associated with strong reductions in neutralizing titers against heterologous viruses. Residues P709, E713 and G725 are antigenically specific for CSFV field strains, and positive selective pressure may influence the cross-neutralization capacity of vaccines. Altogether, these results suggest that positive selection to avoid antibody recognition is a major contributor in CSFV evolution.

## 5. Conclusions

The CSFV-specific conformational epitope containing antigen-specific residues G725 and V738/I738, as well as the CSFV and BDV common conformational epitope comprising antigenic determinant residues P709/L709 and E713, are identified at domain B/C of glycoprotein E2. These epitopes are located in the RBD of E2 and might be involved in the early steps of CSFV infection. Further experiments are necessary to examine this possibility. These findings will further enhance the understanding of the structural and antigenic topography of glycoprotein E2 and will improve the differential diagnosis of porcine pestivirus infections.

## Figures and Tables

**Table 4:** Identification of critical residues of E2-specific mAbs binding by site-directed mutagenesis of CSFV and BDV E2 proteins ^a^.

Mutated Residue ^b^	Strain ^c^	mAbs against E2						
		Domain B/C						Domain A
		CSFV ^d^				BDV		CSFV
		1C7A1	3C1E12	WH220	WH304	WS381	WS384	WH303
None	TD/96	+	+	+	+	+	+	+
	94.4	−	+	−	+	−	+	+
	LPC	−	−	+	+	−	−	+
	Moredun	−	+	−	−	+	+	−
C693A	TD/96	−	−	−	−	−	−	+
C737A		−	−	−	−	−	−	+
P709G	TD/96	+	+	+	+	−	−	+
P709L	TD/96	+	+	+	+	−	±	+
L709P	LPC	−	−	+	−	+	+	+
P709G	Moredun	−	+	−	−	−	−	−
P709L	Moredun	−	+	−	+	−	−	−
E713G	TD/96	+	±	+	+	+	±	+
G713E	LPC	−	+	+	+	−	+	+
E713G	Moredun	−	±	−	−	+	±	−
G725D D725G	TD/96	−	+	+	+	+	+	+
	LPC	+	−	+	+	−	−	+
I738T T738V V738T	TD/96	+	+	−	+	+	+	+
	94.4	−	+	+	+	−	+	+
	LPC	−	−	−	+	−	−	+

^a^ Results were interpreted as positive (+), weak (±) or negative (−) in IFA. The grey highlights indicate mutations that result in a change in binding reactivity compared to the non-mutated E2. ^b^ Mutant E2 proteins are annotated according to the amino acid position of CSFV and the substitution introduced. Refer to Figure 3 for the selection of residues of interest to mutate. ^c^ TD/96, 94.4 and LPC indicated CSFV strain TD/96/TWN of genotype 2.1, 94.4/IL/94/TWN of genotype 3.4 and LPC/AHRI of genotype 1.1, respectively. Moredun indicated BDV Moredun strain of genotype 1.a. ^d^ Derived from CSFV or BDV.

## Data Availability

The data presented in this study are available on request from the corresponding authors.
